# Which patients benefit from physical activity on prescription (PAP)? A prospective observational analysis of factors that predict increased physical activity

**DOI:** 10.1186/s12889-019-6830-1

**Published:** 2019-05-02

**Authors:** Stefan Lundqvist, Mats Börjesson, Maria E. H. Larsson, Åsa Cider, Lars Hagberg

**Affiliations:** 1 Department of Health and Rehabilitation, Unit of Physiotherapy, Institute of Neuroscience and Physiology, Sahlgrenska Academy, University of Gothenburg, Gothenburg, Sweden; 2 Närhälsan Göteborg centrum för fysisk aktivitet, Region Västra Götaland, Gothenburg, Sweden; 3 Department of Food and Nutrition and Sport Science, Faculty of Education, University of Gothenburg, Gothenburg, Sweden; 4 Institute of Neuroscience and Physiology, Sahlgrenska Academy, University of Gothenburg, Gothenburg, Sweden; 5 Sahlgrenska University Hospital/Östra, Gothenburg, Sweden; 6 Närhälsan Research and Development Primary Health Care, Region Västra Götaland, Gothenburg, Sweden; 7 University Health Care Research Center, Faculty of Medicine and Health, Örebro University, Örebro, Sweden

**Keywords:** Physical activity, Metabolic syndrome, Physical activity on prescription, Health behaviour, Correlates of physical activity, Predictive factor

## Abstract

**Background:**

There is robust evidence that regular physical activity (PA) has positive health effects. However, the best PA methods and the most important correlates for promoting PA remain unclear. Physical activity on prescription (PAP) aims to increase the patient’s motivation for and level of PA. This study investigated possible predictive baseline correlates associated with changes in the PA level over a 6-month period of PAP treatment in order to identify the primary care patients most likely to benefit from a PAP intervention.

**Methods:**

The study included 444 patients with metabolic risk factors who were aged 27 to 85 years and physically inactive. The patients received PAP treatment that included individual counseling plus an individually-tailored PA recommendation with a written prescription and individualised structured follow-up for 6 months. Eight baseline correlates of PA were analysed against the PA level at the 6-month follow-up in a predictor analysis.

**Results:**

Five baseline correlates predicted the PA level at the 6-month follow-up: self-efficacy expectations for changing PA; the patient’s preparedness and confidence regarding readiness to change PA; a BMI <  30; and a positive valued physical health. The proportion of patients increasing the PA level and achieving a PA level that was in accordance with public health recommendations was higher with a positive valued baseline correlate. The odds of achieving the recommended PA level increased substantially when 2 to 4 predictive correlates were present. PA levels increased to a greater extent among patients with low PA at baseline than patients with high PA at baseline, especially in combination with 2 to 4 positively-valued correlates (87–95% vs. 62–75%).

**Conclusions:**

This study identified potential predictive correlates of an increased PA level after a 6-month PAP intervention. This contributes to our understanding of PAP and could help individualise PAP support. The proportion of patients with the lowest PA level at baseline increased their PA level in a higher extent (84%) and thus may benefit the most from PAP. These results have clinical implications for behavioural change in those patients having the greatest health gains by increasing their PA level.

**Trial registration:**

ClinicalTrials.gov; NCT03586011. Retrospectively registered on July 17, 2018.

**Electronic supplementary material:**

The online version of this article (10.1186/s12889-019-6830-1) contains supplementary material, which is available to authorized users.

## Background

Physical inactivity is a global health concern [1] that increases the risk of lifestyle-related disorders, including metabolic syndrome (MetS) and premature death [2–4]. MetS typically includes various combinations of overweight, abdominal obesity, insulin resistance, dyslipidaemia, and hypertension [3, 5]. There is a dose-response relationship between physical activity (PA) and health, with the most sedentary and physically inactive patients who increase their PA levels showing more pronounced improvements in health [6–8]. The internationally recommended minimum PA level is 150 min per week of moderate-intensity PA or 75 min per week of vigorous-intensity PA [9].

While the evidence supporting the positive health effects of regular PA is robust [7], many people do not achieve the recommended levels of PA. The best way to promote PA and the factors affecting long-term adherence to a PA program remain unclear [10, 11], and there is a need in clinical practice for studies that evaluate strategies that aim to increase PA [12, 13] and the mechanisms underlying PA behaviour [14]. Specifically, the reasons why PA interventions are effective are not fully understood [10] and the factors or possible correlates that favour successful changes in PA behaviour [14] are not fully elucidated. Sherwood and Jeffery studied the factors associated with increased exercise in PA interventions [15] and found that higher motivation for PA, improved self-efficacy for exercise, enhanced social and environmental support, and tailored interventions for subgroups all helped increase PA.

Such correlates of changes in PA are defined as intervening causal variables that are necessary for creating a cause-effect pathway between different interventions and PA [14, 16, 17]. The mediating variable model proposed by Baranowski et al. [18] suggests that changes in correlates may lead to changes in PA [16, 18, 19], thus identifying why and how treatments exert their effects [20]. Effect modifiers (e.g. age, sex), should also be taken into account. They affect the direction and/or strength of the relationship between an intervention, such as PA, and the outcome [17, 21, 22] and can identify who will be affected by treatments and under what circumstances the treatment will have an effect [20]. Notably, few studies have examined the roles of correlates of PA in experimental PA studies [19], and more research is needed to better understand these possible correlates and PA-related behaviour [23, 24].

Previous studies have identified a number of possible correlates that may affect the outcomes of a PA intervention. These include *readiness to change* based on the principles of the transtheoretical model and the stages of change [25, 26]. The patients readiness to change PA behaviour cannot be taken for granted and the focus will be on that part the patient is most ready to change. *Self-efficacy* is based on social cognitive theory including *self-efficacy expectations*; the belief in capability to perform a behaviour, and *outcome expectations*; the belief that a specific behaviour will lead to a desired outcome [27–29]. *Enjoyment* defines as a positive affective state that reflects feelings such as pleasure and fun [30]. To enhance enjoyment of PA would increase the possibilities for PA changes [10]. *Social support*, based on social cognitive theory [31], is defined as activities that help the individual move toward goals and includes dimensions as social relationships, structure of relationships and functional content of relationships [32]. *Body mass index (BMI)*, and *health-related quality of life* (*HRQOL)* may also affect the outcome of a PA intervention. Previous studies has revealed an inverse association between obesity and PA as well as an impact of HRQOL on PA [22, 33]. Sociodemographic factors such as age, sex, social situation, economic status, education, and smoking may also be important. Studies has shown inversely associations between age in adulthood and PA level, males to be more active than females and that lower levels of education and socio-economic status were associated with less PA [22, 34, 35].

The aim of physical activity on prescription (PAP) is to increase the motivation for PA and the PA level. Swedish PAP studies that are based on an individualized methodology have shown positive effects on PA level, metabolic health, and HRQOL [36–40]. A recent systematic review found that there is a high level of evidence that Swedish PAP increases the PA level [41]. PAP treatment is part of patient-centred care, which takes into account the patient’s knowledge, experience, and needs [42, 43]. PAP treatment includes individual counseling, an individually-tailored PA recommendation with a written prescription, and individualised, structured follow-up [44]. All licensed Swedish professionals in healthcare may use PAP treatment [45]. Further studies are needed to illuminate the effects of Swedish PAP-treatment and the underlying correlates influencing behaviour change in terms of increased PA.

The aim of this study was to explore possible predictive baseline correlates associated with changes in the PA level over a 6-month period of PAP treatment in order to identify the primary care patients most likely to benefit from a PAP intervention.

## Methods

### Study design

The study was a longitudinal prospective observational cohort study of PAP treatment with a 6-month follow-up. The treatment was carried out as part of daily clinical primary care practice and had been in use for several years before the study start. The study design and the PAP intervention has been described previously [40]*.* The study was approved by the Regional Ethical Review Board in Gothenburg, Sweden (Dnr 678–14).

### Study population

**Fig. 1:**
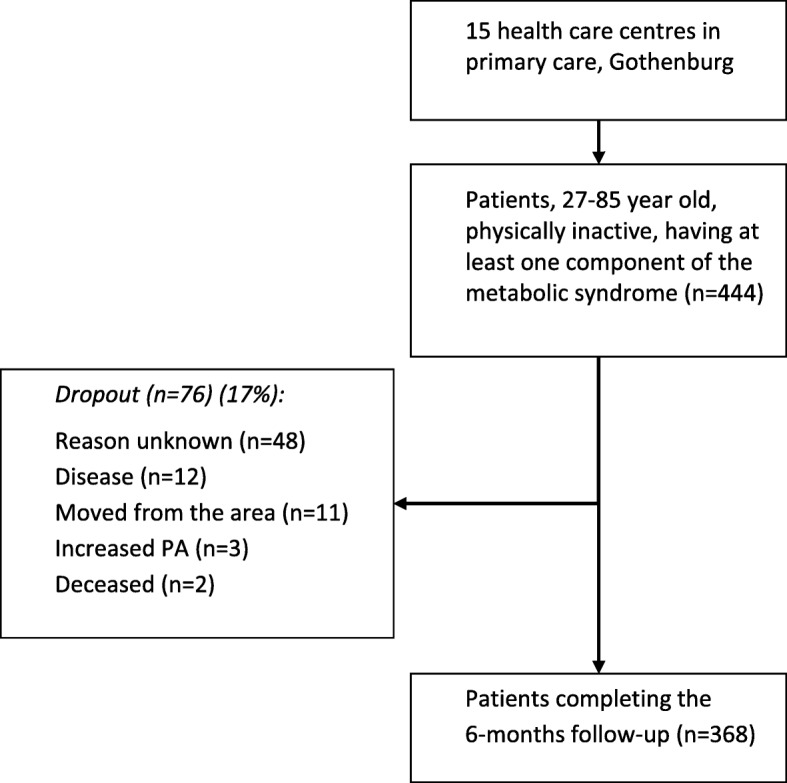
Flow of patients involved in the study

The 444 patients, aged 27–85 years, included in the study were selected as a convenience sample from 15 primary health care centres in Gothenburg, Sweden. The inclusion criteria were: physically inactive according to the internationally recommended minimum PA level [9], having at least one component of MetS present, and receiving PAP treatment. The patients had to understand the Swedish language to complete the questionnaires. The 6-month follow-up was completed by 368 patients, with a dropout rate of 17% (Fig. 1).

The patients, 56% of whom were women, were included prospectively from 2010 to 2014. A majority of the patients were overweight/obese and had hypertension or hyperlipidemia. The mean BMI was 32 kg/m^2^. The majority of patients had two components of MetS, and 61% were using medicines. In the dropout group (*n* = 76, 17%), there was a higher proportion of women, more patients had musculoskeletal disorders, and more had a lower level of HRQOL as measured with the Short Form 36 (SF-36). The characteristics of the study population and the dropouts are presented in Table 1. A more detailed description of the study population has been published previously [40].

**Table 1:** Baseline characteristics of the completer’s and dropout group

Variable	Completer’s (*n* = 368)	Dropout (*n* = 76)	*p* value
Age^a^ – years	57.4 (10.9)	57.6 (13.1)	0.955^e^
Sex^b^			**0.011** ^f^
Female	198 (53.8)	53 (69.7)	
Male	170 (46.2)	23 (30.3)	
Nationality^b^			0.915^f^
Sweden	312 (86**.**0)	62 (84.9)	
Other	51 (14.0)	11 (15.1)	
Social situation^b^			0.144^f^
Single	135 (37.9)	35 (48.6)	
Married/cohabit	205 (57.6)	33 (45.8)	
Other	16 (4.5)	4 (5.6)	
Economic status^b^ – perceived			0.467^f^
Good	213 (59.3)	36 (50.7)	
Neither nor	107 (29.8)	19 (26.8)	
Bad	39 (10.9)	16 (22.5)	
Education^b^			0.117^f^
Elementary grade	69 (19.2)	14 (19.4)	
Upper secondary school	131 (36.4)	36 (50)	
University college	160 (44.4)	22 (30.6)	
Tobacco^b^			0.871^f^
Smokers	34 (9.5)	10 (13.9)	
Non-smokers	229 (63.8)	41 (56.9)	
Ex-smokers	96 (26.7)	21 (29.2)	
Part of metabolic syndrome^b^			
Overweight/Obesity	333 (90.5)	71 (93.4)	0.245^f^
Hyperglycemia	144 (39.1)	30 (39.5)	0.672^f^
Hypertension	293 (79.6)	53 (69.7)	0.117^f^
Hyperlipidemia	212 (57.6)	41 (53.9)	0.801^f^
Other diagnosis			
Mental health, depression	52 (14.1)	13 (17.1)	0.446^f^
Musculoskeletal disorders	58 (15.8)	19 (25)	**0.040** ^f^
Other	155 (42.1)	38 (50)	0.172^f^
Physical activity level^a^, score:			
ACSM/AHA questionnaire	1.7 (1.5)	1.7 (1.4)	0.975^e^
BMI^a^, kg/m^2^	32.0 (5.2)	33.0 (5.8)	0.104^e^
HRQOL SF-36^a^, score:			
Physical component summary	45.7 (9.9)	41.4 (10.8)	**0.001** ^e^
Mental component summary	44.4 (13.1)	40.0 (14.6)	**0.012** ^e^

*ACSM* American College of Sports Medicine, *AHA* American Heart Association, *BMI* body mass index, *HRQOL SF-36* Health Related Quality of Life 36-Item Short Form Health Survey

^a-b^Data are given as ^a^mean (standard deviation) or as ^b^number (percentage)

Difference between follow-up and dropout group. *P*-value was determined by ^e^an independent samples *t*-test or by ^f^a Mann-Whitney U-test

Statistical significance was set at *p* ≤ 0.05. Statistical significant *p*-values are presented in bold numbers

### Intervention

The patient was informed of the possibility to receive PAP treatment and to be included in the study with both written information received in the waiting room and orally by their caregiver. The PAP treatment consisted of an individual counseling, an individually-tailored PA recommendation with a written prescription, and individualised, structured follow-up for 6 months.

Authorised personnel, mainly nurses, who were educated about the effects of PA and the PAP intervention engaged the patient in a patient-centred dialogue about health status and previous and current PA levels. The patient’s preferences for various physical activities, motivation, self-efficacy, and readiness to change PA behaviour were evaluated. An individually dosed PA was agreed upon and written down. The recommended PA was performed by the patient outside the health care system. The most common recommendation was moderate-intensity walking, 30–45 min per episode, and 2–5 times/week to be carried out individually in everyday life. Anthropometrics and blood pressure were measured, blood samples adequate for the MetS analysis were taken and the patient responded to a written questionnaire. The first meeting with the patient lasted 60 to 75 min. During the 6-month intervention period, support was individually structured and involved either revisits or telephone contact. The majority of the patients (80%) visited their PAP responsible nurse 1 to 2 times during the 6-month period, including the 6-month follow-up visit. At the revisit the patient’s motivation, self-efficacy, and readiness to change PA behaviour were re-evaluated and the dose of PA was possibly revised. Each revisit session lasted 30 to 45 min. The measurements, sample collection, and questionnaire were repeated at the 6-month follow-up. The intervention was described in detail previously [40].

### Measurements

The measurements described below were conducted at baseline and at the 6-month follow-up.

#### PA level

The PA level was calculated using a questionnaire based on the public health recommendations of the American College of Sports Medicine (ACSM) and the American Heart Association (AHA) [46]. The questionnaire was used to measure whether the patient achieved the recommended PA level. It was developed by the Lifestyle Intervention Research (LIR) Group at the University of Linköping, Sweden, and included in the working document during the time that new indicator questions regarding PA was evaluated and validated by the Swedish National Board of Health and Welfare [47]. The last 7 days of PA were investigated, and the patient responded to 2 PA questions (ACSM/AHA questionnaire) in which 30 min of moderate-intensity PA per day resulted in 1 point for each day of the week, and 20 min of vigorous-intensity PA per day resulted in 1.7 points for each day of the week. A score ≥ 5 points indicated an adequate PA level according to public health recommendations (≥150 min of moderate-intensity PA/week) [9]. The vigorous-intensity question has been used in previous studies, supporting the construct validity of the measure [48, 49].

#### Correlates of PA change

*Self-efficacy expectations* was measured with *the Self-Efficacy for Exercise Scale (SEES)* [29], with a focus on the patient’s ability to exercise 3 times a week for 20 min in the face of barriers to exercise. The questionnaire has been culturally adopted and translated into Swedish, and it includes nine items (e.g. “The weather was bothering you”, “You had to exercise alone”, “You felt depressed”) that are rated on an ordinal 10 point scale that ranges from 1 (Not confident) to 10 (Very confident). The item scores are summarised and divided by the number of responses, indicating the strength of self-efficacy expectations. The SEES has been tested for use with older adults and older women post-hip fracture; it shows high internal consistency, acceptable reliability, and sufficient to strong evidence for construct and criterion validity [29, 50, 51].

*Outcome expectations* was assessed with *the Outcome Expectations for Exercise-2 Scale (OEE-2)* [28]. The OEE-2 questionnaire is a 13-item measure with 9 positively-worded items (e.g. “Helps me feel less tired”) and 4 negatively-worded items (e.g. “Is something I avoid because it causes me to be short of breath”) that are divided into two subscales: positive OEE and negative OEE. The items are rated on a 5-point Likert scale that ranges from 1 (Strongly agree) to 5 (Strongly disagree). The negative OEE items are reverse-scored, and the numerical ratings for each response are summarised and divided by the number of items. Thus, a highly valued outcome expectation from the patient gives a low total score. The OEE-2 questionnaire was revised in 2005 to include 4 items that concern negative expectations of exercise that are based on qualitative findings [52, 53]. There is some evidence that the OEEE-2 has shown convergent validity, internal consistency, and person and item reliability [28].

*Enjoyment* was measured using *the Physical Activity Enjoyment Scale (PACES)* [54] as modified by Motl et al. [30]. The questionnaire consists of 16 items, 9 of which are positively-worded (e.g. “I think it’s fun”, “It gives me energy”, “It is very pleasant”) and 7 of which are negatively-worded (e.g. “I feel bored”, “I don’t like it”, “It’s frustrating for me”). Each item is rated on a 5-point Likert scale that ranges from 1 (“Does not apply at all”) to 5 (“Truly applies”). The negatively-worded items are reversed-scored, and the responses are added to a score that ranges from 16 to 80. PACES has been tested in 18- to 24-year-old students and adults with functional limitations, and it shows acceptable test-retest reliability, internal consistency, and criterion validity that correlates with physical functioning [54, 55]. The modified PACES has shown satisfactory factorial and construct validity for adolescent girls [30] and invariance for the factor structure, factor loadings and factor variances across time [56].

*Social support* was assessed using *the Social support for exercise scale (SSES)* [32], which includes 13 items that are divided into family and friends sections and measured on a 5-point Likert scale. Of the 13 items, 11 are positively-worded (participation and involvement), and 2 are negatively-worded (rewards and punishments); the items describe social interactions that may be linked to exercise behaviour during the previous 3 months. Responses range from 1 (“None”) to 5 (“Very often”); “Not applicable” is given a score of 1. The item scores are summarised in three subgroups: *Family support – positive, Friend support – positive*, and *Family support – negative*. The *Friend support – negative* subgroup scores were excluded by Sallis et al. because this subgroup did not emerge in the factor analysis. The SSES has acceptable test-retest reliability, high internal consistency, and significant criterion validity that is correlated with a vigorous exercise measure [32].

*The readiness to change PA level* was measured at baseline and included three questions with responses on a 100-mm visual analogue scale (VAS): “How prepared are you to change PA level?”, “How important is it for you to change PA level?”, “How confident are you about succeeding with changing PA level (self-efficacy)?”. The VAS line is anchored at each end with words that describe the minimum and maximum extremes of the dimension that is being measured. The questions are derived from motivational interviewing (MI) and from behaviour change counselling according to Rollnick et al. [25, 57]. A higher value on the VAS indicates an increased readiness to change. The VAS has been used in social and behavioural sciences as a research and clinical tool and is considered to have acceptable reliability and validity [58].

*BMI* was calculated (kg/m^2^) [59] from body weight with the patient in light clothing and without shoes to the nearest 0.1 kg using an electric scale (Carl Lidén AFW D300, Jönköping, Sweden) and from body height measured with the patient in an upright position without shoes to the nearest 0.5 cm using a scale fixed to the wall (Personmått PEM 136, Hultafors, Sweden).

*Health-related quality of life* was assessed with the Swedish version of the Short Form 36 (SF-36 Standard Swedish Version 1.0), which includes 36 questions [60]. It generates 8 health concepts that are grouped into a physical component summary (PCS) and mental component summary (MCS) and that are converted to 0 to 100 points, with higher values representing better HRQL. The SF-36 has shown good to excellent internal consistency and reliability and has been validated in a representative sample of the Swedish population [60].

The following were also recorded: *Age* (years), *sex* (female or male), *social situation* (single or married/cohabiting or other), *economic status* (good; neither good nor bad; bad), *education* (elementary school or high school or university/college), and *smoking* (yes or previous or no).

### Statistical analysis

Interval and ratio data are presented as means and standard deviations (SDs), and nominal and ordinal data as medians and minimum–maximum (min–max or 25–75 percentiles). A per-protocol analysis was used, complemented with an additional ITT analysis, and differences between completer’s and the dropout group were determined by an independent samples *t*-test or by a Mann-Whitney U-test.

Spearman’s rank correlation was used to examine associations between correlates of PA at baseline and PA level at the 6-month follow-up. For each correlate, a mixed linear regression was performed on the ITT population, using PA level at 6-months follow-up as dependent variable and the correlate as fixed independent variable, also including random baseline covariates: age, gender, social situation, economy, education, smoking, and an additional covariate; intervention of care at 6 months. Proceeding with a univariate regression approach using PA level at 6-months follow-up as dependent variable and the correlate as independent variable, a linear fit was done for each model.

The significant correlates from the univariate regression analysis were dichotomised in order to better understand the relationship between baseline versus 6-month variables from a clinical perspective and to evaluate how the intervention could be most effective. This allowed us to perform a predictive analysis with two extreme variants of the correlate values. The correlates were divided into values that were less than or greater than the median value for self-efficacy expectations (SEE), readiness to change–prepared (PREP), readiness to change–confident (CONF), and the SF-36 physical component summary (PCS). The BMI was dichotomised into < 30 versus ≥30. Cut-off values for positively-assessed correlates are presented in Table 2. The PA level at baseline according to the ACSM/AHA questionnaire was dichotomised into low values (< 2 points) versus high values (≥ 2 points) and then combined with the dichotomised correlates of PA in the predictive analysis.

**Table 2:** Cut-points regarding positively assessed correlate values

Baseline correlates	Positive value
SEE, points	≥ 4.77
PREP, mm	> 86
CONF, mm	> 68
BMI	< 30
PCS, points	> 47.06

*SEE* self-efficacy expectations, *PREP* readiness to change – prepared, *CONF* readiness to change – confident, *BMI* body max index, *PCS* physical component summary – SF-36

The dichotomised, statistically significant baseline correlates of PA from the regression analysis were used in a chi-square test for independence. The purpose of this step was to analyse possible predictive correlates to increased PA level (∆-value) and achieved PA level according to the public health recommendation (cut-off value ≥5 points = ≥150 min of moderate-intensity PA/week) at the 6-month follow-up, respectively. The degree of association between variables was measured using the phi coefficient (phi) with Cohen’s criteria of 0.10 for a small effect, 0.30 for a medium effect, and 0.50 for a large effect [61]. Statistical significance was set at *p* ≤ 0.05.

## Results

A total of 368 of the 444 patients, with a mean age of 57 years and 54% female, were retested at the 6-month follow-up (Table 1). Of these patients, 73% (*n* = 270) increased their PA level to some extent, and 42% (*n* = 153) moved from having an inadequate PA level to having a sufficient level according to public health recommendations, as described previously [40]. The results derives from an analysis using per-protocol. An additional ITT analysis (mixed linear regression) including random baseline covariates, was done revealing results that did not make any difference to the conclusions of the article (Additional file 1).

### Factors associated with PA level

There was a significant correlation (*r* = 0.12–0.17) between the following baseline values and the PA level at 6 months: self-efficacy expectations, outcome expectations, enjoyment, readiness to change–PREP, readiness to change–CONF, BMI, physical component summary-SF36 score and baseline PA level (Table 3).

**Table 3:** Spearman’s rank correlation investigating the association between correlates of PA at baseline versus PA level at 6-month follow-up

Correlates of PA (n)	PA level	
	Spearman’s rho (*r*)	*p* value
Self-efficacy expectations (326)	0.14	**0.010**
Outcome expectations (313)	−0.15	**0.007**
Enjoyment (315)	0.12	**0.033**
Social support		
Family-positive (310)	0.07	0.217
Friends-positive (303)	0.07	0.201
Family-negative (327)	0.02	0.790
Readiness to change		
Prepared (348)	0.14	**0.011**
Important (348)	0.04	0.469
Confident (347)	0.17	**0.001**
BMI (357)	−0.14	**0.008**
Physical component summary - SF-36 (344)	0.13	**0.017**
Mental component summary - SF-36 (344)	0.05	0.322
PA level at baseline (361)	0.18	**0.001**
Age (362)	0.03	0.543
Sex (362)	0.06	0.235
Economic status (353)	−0.06	0.225
Social situation (350)	0.07	0.187
Educational level (354)	0.01	0.978
Smoking (353)	0.04	0.443

*PA level* physical activity level according to ACSM/AHA questionnaire, *ACSM* American College of Sports Medicine, *AHA* American Heart Association, *BMI* body mass index, *SF-36* 36-Item Short Form Health Survey

Statistical significance was set at *p* ≤ 0.05. Statistical significant *p*-values are presented in bold numbers

The significant variables from the correlation analysis were used in a univariate regression analysis, which showed significant associations between 6 of the 8 baseline correlates with the PA level at the 6-month follow-up (Table 4). For example, if the self-efficacy expectation score was one point higher at baseline, the PA level score increased by 0.19 points (one point = ≥ 30 min of moderate-intensity PA/day) at the 6-month follow-up. BMI had a significant negative β-coefficient, meaning that if the BMI was 1 kg/cm^2^ higher at baseline, the PA level score decreased by 0.07 points at the 6-month follow-up.

**Table 4:** Univariate regression analysis exploring the relation between correlates of PA at baseline and PA level at 6-month follow-up

Correlates of PA (n)	PA level		
	Unstandardized Coefficient B	R^2^ (adj)	*p* value
Self-efficacy expectations (325)	0.19	0.011	**0.033**
Outcome expectations (312)	−0.56	0.010	0.077
Enjoyment (314)	0.02	0.008	0.118
Readiness to change			
Prepared (347)	0.02	0.013	**0.020**
Confident (346)	0.02	0.027	**0.001**
BMI (356)	−0.07	0.008	**0.045**
Physical component summary - SF-36 (343)	0.04	0.010	**0.033**
PA level at baseline (360)	0.32	0.020	**0.004**

*PA level* physical activity level according to ACSM/AHA questionnaire, *ACSM* American College of Sports Medicine, *AHA* American Heart Association, *BMI* body mass index,

*SF-36* 36-Item Short Form Health Survey

Statistical significance was set at *p* ≤ 0.05. Statistical significant *p*-values are presented in bold numbers

In order to further analyse the relationship between variables at baseline versus at the 6-month follow-up, the significant correlates of PA from the regression analysis were dichotomised into positive and negative values and used in a predictor analysis. A positive value meant that the patient estimated that they had greater self-efficacy to change their PA and were more prepared and more confident in readiness to change PA. A positive value also included a higher estimate of physical health and a BMI <  30. The PA level at baseline was dichotomised into low and high values (< 2 or ≥ 2 points) in the predictive analysis.

### Predictors of increased PA level

Examining one baseline predictor at the time, we found that the proportion of patients increasing PA at the 6-month follow-up was significantly higher with a positive value for readiness to change–CONF, a BMI < 30, or a higher estimated physical health (PCS) (79.0–81.5%, phi 0.12–0.14) (Table 5).

**Table 5:** Percent of patients with increased PA-level (Δ-value) at 6-month follow-up, analysed with 1–2 baseline predictive correlates

Correlate of PA (n)	Increased PA-level (∆ value)			
	% of patients		*p* value^a^	phi coefficient
	Positive values	Negative values		
SEE (170/155)	74.1	72.9	0.804	0.01
PREP (183/164)	77.0	70.7	0.180	0.07
CONF (179/167)	79.9	67.7	**0.010**	0.14
BMI (130/226)	81.5	71.2	**0.031**	0.12
PCS (181/162)	79.0	68.5	**0.027**	0.12
SEE/PREP (109/92)	79.8	75.0	0.414	0.06
SEE/CONF (106/94)	79.2	69.1	0.102	0.12
SEE/BMI (69/106)	82.6	69.8	0.057	0.14
SEE/PCS (95/79)	80.0	69.6	0.114	0.12
PREP/CONF (128/112)	79.7	66.1	**0.017**	0.15
PREP/BMI (62/100)	79.0	64.0	**0.043**	0.16
PREP/PCS (101/80)	83.2	67.5	**0.014**	0.18
CONF/BMI (64/105)	82.8	69.1	**0.004**	0.22
CONF/PCS (102/85)	88.2	68.2	**0.001**	0.24
BMI/PCS (77/113)	84.4	65.5	**0.004**	0.21
	**Low values**	**High values**		
PABL (152/119)	84.0	66.1	**< 0.001**	0.21

*PA-level* physical activity level according to ACSM/AHA questionnaire, *SEE* self-efficacy expectations, *PREP* readiness to change – prepared, *CONF* readiness to change – confident, *BMI* body max index, *PCS* physical component summary – SF-36, *PABL* physical activity at baseline

Cut-points regarding positively assessed values were: SEE ≥4.77 points, PREP > 86 mm, CONF > 68 mm, BMI < 30, PCS ≥ 47.06 points. Cut-point regarding low PABL was < 2 points

^a^*P* values were determined by Chi-square test for independence

Statistical significance was set at *p* ≤ 0.05. Statistical significant *p*-values are presented in bold numbers

Examining two baseline predictors simultaneously allowed us to analyse the associations of 10 combinations of predictors with changes in PA from baseline to the 6-month follow-up. This analysis showed that 79.0–88.2% of patients with two positive predictors were likely to show increased PA compared to 64.0–69.1% of patients with two negative predictors (Table 5). Looking at 3 or 4 baseline predictors simultaneously showed a similar trend regarding increased PA as when two baseline predictors were used, but this was not significant (data shown in Additional file 2). The correlations for significant values were small (phi 0.15–0.24).

Patients with a low PA level at baseline (< 2 points) increased their PA at the 6-month follow-up to a greater extent than patients with a high PA level at baseline (≥ 2 points) (*p* < 0.001) (Table 5). Strikingly, at the 6-month follow-up, subjects with a low PA level at baseline plus 1 to 3 positive baseline predictors were even more likely to have increased their PA (87–95%, *p* ≤ 0.002) (data shown in Additional file 3).

### Predictors for achieving the recommended PA level

The proportion of patients achieving a PA level ≥ 5 points, which is the recommended PA level, was higher with a significantly positive value for readiness to change–CONF and a BMI < 30 at baseline (48.3–50.4%, phi 0.11–0.12) (Table 6).

**Table 6:** Percent of patients with reached PA-level ≥ 5p at 6-month follow-up, analysed with 1–2 baseline predictive correlates

Correlate of PA (n)	Reached PA-level (≥ 5p)			
	% of patients		*p* value^a^	phi coefficient
	Positive values	Negative values		
SEE (171/115)	44.4	38.7	0.294	0.06
PREP (183/165)	47.0	37.6	0.076	0.10
CONF (180/167)	48.3	35.9	**0.019**	0.12
BMI (131/226)	50.4	39.4	**0.043**	0.11
PCS (182/162)	46.2	37.7	0.111	0.09
SEE/PREP (109/92)	49.5	38.0	0.102	0.12
SEE/CONF (107/94)	47.7	34.0	**0.050**	0.14
SEE/BMI (70/106)	54.3	37.7	**0.031**	0.16
SEE/PCS (96/79)	47.9	34.2	0.067	0.14
PREP/CONF (128/112)	49.2	33.0	**0.011**	0.16
PREP/BMI (62/100)	53.2	32.0	**0.007**	0.21
PREP/PCS (101/80)	50.5	33.8	**0.024**	0.17
CONF/BMI (65/105)	50.8	28.6	**0.004**	0.22
CONF/PCS (103/85)	51.5	31.8	**0.007**	0.20
BMI/PCS (78/113)	48.7	31.9	**0.019**	0.17
	**Low values**	**High values**		
PABL (70/85)	38.7	47.2	0.101	0.09

*PA-level* physical activity level according to ACSM/AHA questionnaire, *SEE* self-efficacy expectations, *PREP* readiness to change – prepared, *CONF* readiness to change – confident, *BMI* body max index, *PCS* physical component summary – SF-36, *PABL* physical activity at baseline. Cut-points regarding positively assessed values were: SEE ≥4.77 points, PREP > 86 mm, CONF > 68 mm, BMI < 30, PCS ≥ 47.06 points. Cut-point regarding low PABL was < 2 points

^a^*P* values were determined by Chi-square test for independence

Statistical significance was set at *p* ≤ 0.05. Statistical significant *p*-values are presented in bold numbers

The proportion of patients reaching the recommended PA level overall was higher with two positive valued baseline predictors (47.7–54.3%) compared to 28.6–34% for patients with two negative valued baseline predictors. The majority of the values were significant, with a small correlation value (phi 0.14–0.22) (Table 6). Analysis of 3 baseline predictors simultaneously (data shown in Additional file 4) gave similar results as an analysis using 2 predictors, while an analysis of 4 baseline predictors simultaneously increased the correlation value (phi 0.33) between the positive and negative predictors in terms of achieving the recommended PA level.

Patients with a high PA level at baseline (≥ 2 points) seemed to reach a PA level ≥ 5 points at the 6-month follow-up to a greater extent than patients with a low PA level at baseline (< 2 points) (47.2% vs. 38.7%); however, this result was not significant (*p* = 0.101) (Table 6). A high PA level at baseline plus 1 to 3 positive valued baseline predictors resulted in a more frequently reached PA level ≥ 5p at 6-month follow-up (50–57%, *p* = ≤0.026) (data shown in Additional file 5).

## Discussion

This study’s main finding was that a number of baseline factors were associated with the PA level at the 6-month follow-up after the PAP intervention. These predictive factors included greater self-efficacy expectations, more preparedness and confidence in terms of one’s readiness to change, a BMI < 30 and a positive value for a measure of physical health at baseline. The proportion of patients with these predictors were higher in the group that increased PA after PAP. In addition, if 2 to 4 of these factors were present, the likelihood of reaching a sufficient PA level (according to public health recommendations) increased significantly. These findings highlight potential predictors of PAP that can lead to an increased PA level at a 6-month follow-up. Considering these predictors at an early stage of the intervention could improve individualised support of the patient during the behavioural change process.

This study investigated which primary care patients are most likely to benefit from PAP treatment and found that the proportion of patients with low PA at baseline increased their PA level in a higher extent compared to patients with high PA at baseline, especially in combination with positive valued predictors (maximum of 95% vs. 65%). These results suggest that patients with the lowest PA levels benefit most from PAP. Considering the dose-response relationship between sedentary time, PA and health and the more pronounced health gain for those changing from sedentary/low PA level [7, 8, 62], this could have important clinical implications.

One strength of this study was its size. The included patients represent a major patient group that may be suitable for receiving PAP, and the PAP treatment was executed in a daily clinical primary care setting. Even if a majority of patients had two components of MetS and were treated with medication, showing higher complexity, the majority of the patients increased their PA level and health parameters during the 6-month PAP treatment [40]. These results indicate that the PAP treatment may be suitable for use in broader groups of patients with medical conditions that range from less severe to more severe, but this awaits further study. Another strength of the study is that the individualised PAP intervention commonly resulted in a PA recommendation to be carried out in everyday life (e.g. walking), which is suitable for the most physically inactive patients. These patients are, from a health perspective, the most vulnerable and have the most to gain from an increased PA level [6–8].

PA is a complex behaviour, and it is complicated to measure how factors such as PA category, type, frequency, duration, intensity, and purpose affect PA measurement and outcome [8, 63]. PA behaviour is also affected by diverse factors at the individual, social, psychological, behavioural, environmental, and policy levels [22, 64]. Barnett et al. [34] found that adults increased their PA in order to improve their health and well-being and to establish new daily routines with the opportunity for social interactions and personal challenges. PA behaviour is also affected by genetics and controlled by neural and situational processes which are difficult to measure and control [18, 65]. In light of the fact that PA behaviour is affected by many variables and is complicated to measure, it is not surprising that there are small correlation (r^2^) values between the correlates of PA and the PA level [18, 21, 50].

It has been argued that there are likely to be errors when correlates of PA are measured using multiple regression analyses because the correlates are often internal, psychological variables [21]. Thus, the effect of the correlate tends to be underestimated while the effect of the independent variable on the dependent variable tends to be overestimated. Hansen et al. [65] argue that behavioural interventions only have indirect effects i.e. they may change the predisposing and enabling factors that lead to behaviour but not the behaviour itself, resulting in lower correlation values. Although the present study showed overall small r^2^ values, it could take into consideration potential predictors of increased PA level at the 6-month follow-up of the PAP treatment.

In the dichotomised predictor analysis, a positive value for confidence in readiness to change and BMI < 30 were associated with an increased PA level and with a higher degree of fulfilment of the PA recommendations, respectively. Confidence in readiness to change emanates from the concept of self-efficacy that, in previous research, has been shown to be one of the most important correlates of PA for adults [16, 22]. This study found significant values for VAS-estimated confidence/self-efficacy but not for the Self-Efficacy for Exercise (SEES) questionnaire in the dichotomised analysis. The reason that the SEES questionnaire did not show significant values could be due to the use of the word *exercise* instead of *physical activity* and the predetermined level of exercise as 20 min, three times per week. This could have complicated estimation of PA by the patient. In contrast, marking readiness to change PA on a VAS seems more feasible.

It is well known that BMI has an inverse association with PA, and while PA may be a determinant of obesity, obesity may also be a determinant of PA [22, 66]. In a previous analysis of this study group [40], the study group showed a decrease in BMI at the 6-month follow-up of the PAP treatment. Here, in the dichotomised analysis, an increased PA level and fulfilment of PA recommendations at 6 months could be predicted based on a baseline BMI < 30. These results support both determinant theories i.e. that PA is a determinant of obesity and vice versa. In this study the patients had a mean BMI value of 32 and 73% of the patients increased their PA level in some extent indicating that patients with BMI > 30 benefits from PAP-treatment. However, further subgroup analysis is needed to investigate the content and amount of PAP-support for patients with a BMI > 30. As in previous research on HRQOL, our results indicate that a positive value for physical health as measured on the SF-36 plays a role in predicting an increased PA level at the 6-month follow-up [67, 68].

The simultaneous use of 2 to 4 baseline predictors in the analysis (with fewer patients in the 4-predictor analysis) showed a clearer PA trend for patients with positive vs. negative values, especially regarding the fulfilment of PA recommendations. In the study group, 73% of the patients increased their PA level to some extent, and 42% achieved the PA recommendations at the 6-month follow-up. For many patients, reaching a PA level ≥ 5 points was a major step, and the results showed that having 2 to 4 predictors with positive values at baseline increased the possibility of predicting achievement of the PA recommendation. Sherwood et al. [15] stated that we have to incorporate multiple determinants of PA that reflect the complexity of predicting exercise behaviour, and Trost et al. [69] argued that longitudinal and intervention studies are needed to infer causal relationships. To our knowledge, there is no research that assesses multiple predictive correlates with increased PA level in PAP interventions. The present study thus contributes to the knowledge about predictors of increased PA at a 6-month follow-up of PAP treatment. Further studies are, however, needed in the research field of predicting correlates.

### Limitations

This study has some limitations [40]. The dropout rate of 17% could influence the interpretation of results as it may have introduced selection bias. Nevertheless, the study was a “daily clinical work” survey, and the dropout rate was about as expected for this type of study. As in previous published article [40] a per-protocol analysis was used rather than an intention to treat analysis (ITT), which could increase the risk of bias. However, ITT has been criticised for increasing the risk of attributing biased characteristics. In this article, an additional ITT analysis was done revealing results that did not make any difference to our conclusions. The lack of a control group could complicate the interpretation of the result but there is an overall discussion about the limitations with RCT’s due to the possible lack of external validity. The study population was included non-consecutively, which may increase the risk of selection bias. However, PAP treatment is patient-centred and takes into account the patient’s attitude about changing their PA level, and PAP probably has the most potential to help patients who have been thinking of changing their habits. The use of self-reported measures increases the risk of over- or underestimating the items in questionnaires and to be affected by recall and response bias [70]. These measures are still frequently used due to their practicality, general acceptance and ability to collect data from a large number of patients at low cost [71]. To choose a measurement most appropriate for the dimension of behaviour of interest is essential [72] and the instruments for correlate measurement used in the article has previously been reliability and validity tested.

### Clinical implications

The PAP intervention in this study is most likely to increase the PA levels of patients who have a low PA at baseline, who have confidence in their readiness to change their PA level, who have better estimated physical health, or who have a BMI < 30. Identifying these possible predictive correlates at an early stage of the PAP intervention offers clinicians an opportunity to support the patient during the behavioural change process and to individualise PAP treatment in order to increase the patient’s PA level.

## Conclusions

This study identified potential predictive correlates of an increased PA level after a 6-month PAP intervention. This may help improve the individualisation of PAP support.

The results indicated that the proportion of patients with the lowest PA levels increased their PA level in a higher extent (84%), primarily benefiting from the PAP intervention. These results have clinical implications for professionals who work to promote behavioural changes in patients who can potentially improve their health by increasing their PA level.

## Additional files

Additional file 1: Regression analysis based on the ITT population including covariates and adding intervention contact. (PDF 79 kb)

Additional file 2: Percent of patients with increased PA-level (Δ-value) at 6-month follow-up, analyzed with 3–4 baseline predictive correlates. (PDF 205 kb)

Additional file 3: Percent of patients with increased PA-level (Δ-value) at 6-month follow-up, analyzed with low vs. high PA at baseline and 1–3 baseline predictive correlates. (PDF 218 kb)

Additional file 4: Percent of patients with reached PA-level ≥ 5p at 6-month follow-up, analyzed with 3–4 baseline predictive correlates. (PDF 206 kb)

Additional file 5: Percent of patients with reached PA-level ≥ 5p at 6-month follow-up, analyzed with low vs. high PA at baseline and 1–3 baseline predictive correlates. (PDF 261 kb)

## Abbreviations

ACSM: American College of Sports Medicine
AHA: American Heart Association
BMI: Body mass index
CONF: Readiness to change–confident
HCC: Health care centre
HRQOL: Health-related quality of life
ITT: Intention to treat analysis
LIR: Lifestyle Intervention Research
MetS: Metabolic syndrome
MI: Motivational interviewing
OEE-2: Outcome Expectations for Exercise-2 Scale
PA: Physical activity
PABL: Physical activity at baseline
PACES: Physical Activity Enjoyment Scale
PAP: Physical activity on prescription
PCS: SF-36 physical component summary
PREP: Readiness to change–prepared
RCT: Randomised controlled trial
SEE: Self-efficacy expectations
SEES: Self-Efficacy for Exercise Scale
SF-36: 36-Item Short Form Health Survey
SSES: Social support for exercise scale
VAS: Visual analogue scale

## References

[1] Das P, Horton R (2012). Rethinking our approach to physical activity. Lancet..

[2] World Health Organization. Global health risks: mortality and burden of disease attributable to selected major risks: World Health Organization; 2009.

[3] Edwardson CL, Gorely T, Davies MJ, Gray LJ, Khunti K, Wilmot EG (2012). Association of sedentary behaviour with metabolic syndrome: a meta-analysis. PLoS One.

[4] Forouzanfar MH, Alexander L, Anderson HR, Bachman VF, Biryukov S, Brauer M (2015). Global, regional, and national comparative risk assessment of 79 behavioural, environmental and occupational, and metabolic risks or clusters of risks in 188 countries, 1990–2013: a systematic analysis for the global burden of disease study 2013. Lancet.

[5] Grundy SM, Brewer HB, Cleeman JI, Smith SC, Lenfant C (2004). Definition of metabolic syndrome: report of the National Heart, Lung, and Blood Institute/American Heart Association conference on scientific issues related to definition. Circulation..

[6] Physical Activity Guidelines Advisory Committee report, 2008 (2009). To the Secretary of Health and Human Services. Part A: executive summary. Nutr Rev.

[7] Lee IM, Shiroma EJ, Lobelo F, Puska P, Blair SN, Katzmarzyk PT (2012). Effect of physical inactivity on major non-communicable diseases worldwide: an analysis of burden of disease and life expectancy. Lancet..

[8] Wen CP, Wai JP, Tsai MK, Yang YC, Cheng TY, Lee MC (2011). Minimum amount of physical activity for reduced mortality and extended life expectancy: a prospective cohort study. Lancet..

[9] World Health Organization. WHO Guidelines Approved by the Guidelines Review Committee. Global Recommendations on Physical Activity for Health. Geneva: World Health Organization; 2010.

[10] Hagberg LA, Lindahl B, Nyberg L, Hellenius ML (2009). Importance of enjoyment when promoting physical exercise. Scand J Med Sci Sports.

[11] Joy E, Blair SN, McBride P, Sallis R (2013). Physical activity counselling in sports medicine: a call to action. Br J Sports Med.

[12] Persson G, Brorsson A, Ekvall Hansson E, Troein M, Strandberg EL (2013). Physical activity on prescription (PAP) from the general practitioner’s perspective – a qualitative study. BMC Fam Pract.

[13] Sallis R, Franklin B, Joy L, Ross R, Sabgir D, Stone J (2015). Strategies for promoting physical activity in clinical practice. Prog Cardiovasc Dis.

[14] Bauman AE, Sallis JF, Dzewaltowski DA, Owen N (2002). Toward a better understanding of the influences on physical activity: the role of determinants, correlates, causal variables, mediators, moderators, and confounders. Am J Prev Med.

[15] Sherwood NE, Jeffery RW (2000). The behavioral determinants of exercise: implications for physical activity interventions. Annu Rev Nutr.

[16] Biddle S, Mutrie N, Gorely T. Psychology of physical activity : determinants, well-being and interventions 2015.

[17] Breitborde NJ, Srihari VH, Pollard JM, Addington DN, Woods SW (2010). Mediators and moderators in early intervention research. Early intervention in psychiatry.

[18] Baranowski T, Anderson C, Carmack C (1998). Mediating variable framework in physical activity interventions. How are we doing? How might we do better?. Am J Prev Med.

[19] Rhodes RE, Pfaeffli LA (2010). Mediators of physical activity behaviour change among adult non-clinical populations: a review update. Int. J. Behav. Nutr. Phys. Act..

[20] Kraemer HC, Wilson GT, Fairburn CG, Agras WS (2002). Mediators and moderators of treatment effects in randomized clinical trials. Arch Gen Psychiatry.

[21] Baron RM, Kenny DA (1986). The moderator-mediator variable distinction in social psychological research: conceptual, strategic, and statistical considerations. J Pers Soc Psychol.

[22] Bauman AE, Reis RS, Sallis JF, Wells JC, Loos RJ, Martin BW (2012). Correlates of physical activity: why are some people physically active and others not?. Lancet..

[23] Fappa E, Yannakoulia M, Pitsavos C, Skoumas I, Valourdou S, Stefanadis C (2008). Lifestyle intervention in the management of metabolic syndrome: could we improve adherence issues?. Nutrition (Burbank, Los Angeles County, Calif).

[24] Kirk AF, Barnett J, Mutrie N (2007). Physical activity consultation for people with type 2 diabetes: evidence and guidelines. Diabet Med.

[25] Rollnick S, Mason P, Butler C (1999). Health behavior change: a guide for practitioners.

[26] Prochaska JO, DiClemente CC (1983). Stages and processes of self-change of smoking: toward an integrative model of change. J Consult Clin Psychol.

[27] Bandura A (1977). Self-efficacy: toward a unifying theory of behavioral change. Psychol Rev.

[28] Resnick B (2005). Reliability and validity of the outcome expectations for exercise Scale-2. J Aging Phys Act.

[29] Resnick B, Jenkins LS (2000). Testing the reliability and validity of the self-efficacy for exercise scale. Nurs Res.

[30] Motl RW, Dishman RK, Saunders R, Dowda M, Felton G, Pate RR (2001). Measuring enjoyment of physical activity in adolescent girls. Am J Prev Med.

[31] Bandura A (1986). Social foundations of thought and action : a social cognitive theory.

[32] Sallis JF, Grossman RM, Pinski RB, Patterson TL, Nader PR (1987). The development of scales to measure social support for diet and exercise behaviors. Prev Med.

[33] Allender S, Hutchinson L, Foster C (2008). Life-change events and participation in physical activity: a systematic review. Health Promot Int.

[34] Barnett I, Guell C, Ogilvie D (2012). The experience of physical activity and the transition to retirement: a systematic review and integrative synthesis of qualitative and quantitative evidence. Int. J. Behav. Nutr. Phys. Act..

[35] Harris TJ, Owen CG, Victor CR, Adams R, Cook DG (2009). What factors are associated with physical activity in older people, assessed objectively by accelerometry?. Br J Sports Med.

[36] Kallings LV, Sierra Johnson J, Fisher RM, Faire U, Stahle A, Hemmingsson E (2009). Beneficial effects of individualized physical activity on prescription on body composition and cardiometabolic risk factors: results from a randomized controlled trial. Eur J Cardiovasc Prev Rehabil.

[37] Leijon ME, Bendtsen P, Nilsen P, Festin K, Stahle A (2009). Does a physical activity referral scheme improve the physical activity among routine primary health care patients?. Scand J Med Sci Sports.

[38] Olsson SJ, Börjesson M, Ekblom-Bak E, Hemmingsson E, Hellénius ML, Kallings LV (2015). Effects of the Swedish physical activity on prescription model on health-related quality of life in overweight older adults: a randomised controlled trial. BMC Public Health.

[39] Rodjer L, HJ I, Borjesson M (2016). Physical activity on prescription (PAP): self-reported physical activity and quality of life in a Swedish primary care population, 2-year follow-up. Scand J Prim Health Care.

[40] Lundqvist S, Borjesson M, Larsson ME, Hagberg L, Cider A (2017). Physical Activity on Prescription (PAP), in patients with metabolic risk factors. A 6-month follow-up study in primary health care. PLoS One.

[41] Börjesson M, Arvidsson D, Blomqvist Å, Daxberg E-L, Jonsdottir IH, Lundqvist S, et al. Efficacy of the Swedish model for physical activity on prescription [Effektivitet av den svenska modellen för fysisk aktivitet på recept (FaR)]. Göteborg: Västra Götalandsregionen, Sahlgrenska Universitetssjukhuset, HTA-centrum, 2018. Regional activity based HTA 2018:100.

[42] Balint M (1955). The doctor, his patient, and the illness. Lancet..

[43] Dwamena F, Holmes-Rovner M, Gaulden CM, Jorgenson S, Sadigh G, Sikorskii A (2012). Interventions for providers to promote a patient-centred approach in clinical consultations. The Cochrane database of systematic reviews.

[44] Kallings LV (2008). Physical activity on prescription : studies on physical activity level, adherence and cardiovascular risk factors.

[45] Nationella riktlinjer : remissversion : prevention och behandling vid ohälsosamma levnadsvanor. [Stockholm]: Socialstyrelsen; 2017.

[46] Haskell WL, Lee IM, Pate RR, Powell KE, Blair SN, Franklin BA (2007). Physical activity and public health: updated recommendation for adults from the American College of Sports Medicine and the American Heart Association. Med Sci Sports Exerc.

[47] Hagströmer M, Wisén A, Hassmén P. Bedöma och utvärdera fysisk aktivitet. FYSS Fysisk aktivitet i sjukdomsprevention och sjukdomsbehandling 2015. 26574808

[48] Sallis JF, Hovell MF, Hofstetter CR (1992). Predictors of adoption and maintenance of vigorous physical activity in men and women. Prev Med.

[49] Sallis JF, Hovell MF, Hofstetter CR, Barrington E (1992). Explanation of vigorous physical activity during two years using social learning variables. Soc Sci Med.

[50] Resnick B, Luisi D, Vogel A, Junaleepa P (2004). Reliability and validity of the self-efficacy for exercise and outcome expectations for exercise scales with minority older adults. J Nurs Meas.

[51] Resnick B, Orwig D, Zimmerman S, Hawkes W, Golden J, Werner-Bronzert M (2006). Testing of the SEE and OEE post-hip fracture. West J Nurs Res.

[52] Resnick B, Magaziner J, Orwig D, Zimmerman S (2002). Evaluating the components of the exercise plus program: rationale, theory and implementation. Health Educ Res.

[53] Resnick B, Vogel A, Luisi D (2006). Motivating minority older adults to exercise. Cultural diversity & ethnic minority psychology.

[54] Kendzierski D, DeCarlo KJ. Physical activity enjoyment scale: two validation studies. Journal of Sport & Exercise Psychology. 1991;13(1):50–64.

[55] Murrock CJ, Bekhet A, Zauszniewski JA (2016). Psychometric evaluation of the physical activity enjoyment scale in adults with functional limitations. Issues in mental health nursing.

[56] Dishman RK, Motl RW, Saunders R, Felton G, Ward DS, Dowda M (2005). Enjoyment mediates effects of a school-based physical-activity intervention. Med Sci Sports Exerc.

[57] Stott NCH, Rollnick S, Rees MR, Pill RM, Anwar A, Besch S (1995). Innovation in clinical method: diabetes care and negotiating skills. Fam Pract.

[58] McCormack HM, Horne DJ, Sheather S (1988). Clinical applications of visual analogue scales: a critical review. Psychol Med.

[59] Keys A, Fidanza F, Karvonen MJ, Kimura N, Taylor HL (1972). Indices of relative weight and obesity. J Chronic Dis.

[60] Sullivan M, Karlsson J, Ware JE (1995). The Swedish SF-36 health survey--I. evaluation of data quality, scaling assumptions, reliability and construct validity across general populations in Sweden. Soc Sci Med.

[61] Cohen J (1988). Statistical power analysis for the behavioral sciences.

[62] Ekelund U, Steene-Johannessen J, Brown WJ, Fagerland MW, Owen N, Powell KE (2016). Does physical activity attenuate, or even eliminate, the detrimental association of sitting time with mortality? A harmonised meta-analysis of data from more than 1 million men and women. Lancet..

[63] Caspersen CJ, Powell KE, Christenson GM (1985). Physical activity, exercise, and physical fitness: definitions and distinctions for health-related research. Public health reports (Washington, DC : 1974).

[64] Andreasson J, Tugetam Å, Bergman P (2016). Keeping death at bay through health negotiation: older adults’ understanding of health and life within gym and fitness culture. Activities, Adaptation and Aging.

[65] Hansen WB, McNeal RB (1996). The law of maximum expected potential effect: constraints placed on program effectiveness by mediator relationships. Health Educ Res.

[66] Ekelund U, Brage S, Besson H, Sharp S, Wareham NJ (2008). Time spent being sedentary and weight gain in healthy adults: reverse or bidirectional causality?. Am J Clin Nutr.

[67] Rejeski WJ, Brawley LR, Ambrosius WT, Brubaker PH, Focht BC, Foy CG (2003). Older adults with chronic disease: benefits of group-mediated counseling in the promotion of physically active lifestyles. Health Psychol.

[68] Rejeski WJ, Brawley LR, Shumaker SA (1996). Physical activity and health-related quality of life. Exerc Sport Sci Rev.

[69] Trost SG, Owen N, Bauman AE, Sallis JF, Brown W (2002). Correlates of adults' participation in physical activity: review and update. Med Sci Sports Exerc.

[70] Adamo KB, Prince SA, Tricco AC, Connor-Gorber S, Tremblay M (2009). A comparison of indirect versus direct measures for assessing physical activity in the pediatric population: a systematic review. Int J Pediatr Obes.

[71] Sallis JF, Saelens BE (2000). Assessment of physical activity by self-report: status, limitations, and future directions. Res Q Exerc Sport.

[72] Linton MJ, Dieppe P, Medina-Lara A (2016). Review of 99 self-report measures for assessing well-being in adults: exploring dimensions of well-being and developments over time. BMJ Open.

