# Supplementary material for: Which patients benefit from physical activity on prescription (PAP)? A prospective observational analysis of factors that predict increased physical activity
Source: BMC Public Health. 2019 May 2;19:482. doi: 10.1186/s12889-019-6830-1 (PMC6498468; doi:10.1186/s12889-019-6830-1)
Supplement: Supplementary file 1 — Regression analysis based on the ITT population including covariates and adding intervention contact. (PDF 79 kb) [file 12889_2019_6830_MOESM1_ESM.pdf]

Additional file 1

Additional regression analyses including covariates and adding intervention contact

We have made an additional regression analysis base on the ITT population. Using a mixed linear regression model, PA at six months is used as dependent factor and each predictor as independent fixed factor. The following random factors are included: age, sex, social situation, economy, education, smoking. The covariate intervention of care at 6 months is also included as a random factor after suggestion from reviewers.

Results are presented below and shows that Self-efficacy expectations, Readiness to change-Confident, BMI, and Physical component summary SF-36 are significant predictors. Hence, we see that these results will not change any of our previous analyses.

Regression analysis based on the ITT population including covariates and adding intervention contact	
**Independent variable (baseline correlates) (n)**	***p* value**
Self-efficacy expectation (299)	**0.047**
Outcome expectation (286)	0.07
Enjoyment (291)	0.13
Social support	
Family-positive (282)	0.11
Family-negative (300)	0.87
Friend-positive (280)	0.65
Readiness to change	
Prepared (318)	0.09
Important (348)	0.87
Confident (318)	**0.002**
BMI (324)	**0.016**
Physical component summary SF-36 (314)	**0.028**
Mental component summary SF-36 (314)	0.88

Mixed model using dependent variable PA level at 6 months
Independent variables; correlates of PA in a univariate fashion (fixed factor)
Baseline covariates; age, sex, social situation, economy, education, smoking and additionally intervention of care at 6 months (random factors)
Statistical significance was set at $p \leq 0.05$
