# Supplementary material for: Which patients benefit from physical activity on prescription (PAP)? A prospective observational analysis of factors that predict increased physical activity
Source: BMC Public Health. 2019 May 2;19:482. doi: 10.1186/s12889-019-6830-1 (PMC6498468; doi:10.1186/s12889-019-6830-1)
Supplement: Supplementary file 2 — Percent of patients with increased PA-level (Δ-value) at 6-month follow-up, analyzed with 3–4 baseline predictive correlates. (PDF 205 kb) [file 12889_2019_6830_MOESM2_ESM.pdf]

Additional file 2 Percent of patients with increased PA-level (Δ-value) at 6-month follow-up, analysed with 3-4 baseline predictive correlates

**Correlate of PA (n)**	**Increased PA-level (Δ value)**			
	**% of patients**		***p* value**[a]	**phi coefficient**
	**Positive values**	**Negative values**		
CONF/PREP/SEE (82/68)	80.5	72.1	0.224	0.10
CONF/PREP/SEE/PCS (47/36)	87.2	72.2	0.086	0.19

PA-level physical activity level according to ACSM/AHA questionnaire, *CONF* readiness to change confident, *PREP* readiness to change – prepared, *SEE* self-efficacy expectations, *PCS* physical component summary – SF-36

Cut-points regarding positively assessed values were: CONF > 68 mm, PREP > 86 mm, SEE ≥ 4.77 points, PCS ≥ 47.06 points

[a]*P* values were determined by Chi-square test for independence

Statistical significance was set at $p \leq 0.05$
