# Supplementary material for: Which patients benefit from physical activity on prescription (PAP)? A prospective observational analysis of factors that predict increased physical activity
Source: BMC Public Health. 2019 May 2;19:482. doi: 10.1186/s12889-019-6830-1 (PMC6498468; doi:10.1186/s12889-019-6830-1)
Supplement: Supplementary file 3 — Percent of patients with increased PA-level (Δ-value) at 6-month follow-up, analyzed with low vs. high PA at baseline and 1–3 baseline predictive correlates. (PDF 218 kb) [file 12889_2019_6830_MOESM3_ESM.pdf]

Additional file 3 Percent of patients with increased PA-level (Δ-value) at 6-month follow-up, analysed with low vs. high PA at baseline and 1-3 baseline predictive correlates

**Correlate of PA (n)**	**Increased PA-level (Δ value)**			
	**% of patients**		***p* value[a]**	**phi coefficient**
	**Low/Positive values**	**High/Negative values**		
PABL/SEE (71/48)	86.6	65.8	**0.002**	0.25
PABL/PREP (79/48)	86.8	60.8	**<0.001**	0.30
PABL/CONF (79/46)	90.8	58.2	**<0.001**	0.38
PABL/PCS (80/48)	87.0	58.5	**<0.001**	0.32
PABL/BMI (57/70)	87.7	61.4	**<0.001**	0.28
PABL/CONF/PREP (59/27)	90.8	54.0	**<0.001**	0.42
PABL/CONF/PREP/SEE (40/19)	95.2	65.5	**0.001**	0.39
	**Low/Negative values**	**High/Positive values**		
PABL/SEE (65/55)	79.3	62.5	**0.017**	0.18
PABL/PREP (68/62)	80.0	67.4	0.058	0.14
PABL/CONF (67/64)	76.1	69.6	0.322	0.07
PABL/PCS (63/63)	78.8	70.8	0.235	0.09
PABL/BMI (91/49)	81.3	75.4	0.355	0.07
PABL/CONF/PREP (47/43)	75.8	68.3	0.347	0.08
PABL/CONF/PREP/SEE (30/26)	76.9	65.0	0.234	0.13

PA-level physical activity level according to ACSM/AHA questionnaire, *PABL* physical activity at baseline, *SEE* self-efficacy expectations, *PREP* readiness to change – prepared, *CONF* readiness to change – confident, *PCS* physical component summary - SF-36, *BMI* body mass index

[a]*P* values were determined by Chi-square test for independence

Statistical significance was set at $p \leq 0.05$
