# Supplementary material for: Which patients benefit from physical activity on prescription (PAP)? A prospective observational analysis of factors that predict increased physical activity
Source: BMC Public Health. 2019 May 2;19:482. doi: 10.1186/s12889-019-6830-1 (PMC6498468; doi:10.1186/s12889-019-6830-1)
Supplement: Supplementary file 4 — Percent of patients with reached PA-level ≥ 5p at 6-month follow-up, analyzed with 3–4 baseline predictive correlates. (PDF 206 kb) [file 12889_2019_6830_MOESM4_ESM.pdf]

Additional file 4 Percent of patients with reached PA-level ≥ 5p at 6-month follow-up, analysed with 3-4 baseline predictive correlates

**Correlate of PA (n)**	**Reached PA-level (≥ 5p)**			
	**% of patients**		**p value[a]**	**phi coefficient**
	**Positive values**	**Negative values**		
CONF/PREP/SEE (82/68)	50.0	33.8	**0.046**	0.16
CONF/PREP/SEE/PCS (47/36)	55.3	22.2	**0.002**	0.33

PA-level physical activity level according to ACSM/AHA questionnaire, *CONF* readiness to change confident, *PREP* readiness to change – prepared, *SEE* self-efficacy expectations, *PCS* physical component summary – SF-36.
Cut-points regarding positively assessed values were: CONF > 68 mm, PREP > 86 mm, SEE ≥ 4.77 points, PCS ≥ 47.06 points
[a]*P* values were determined by Chi-square test for independence
Statistical significance was set at $p \leq 0.05$
