# Supplementary material for: Which patients benefit from physical activity on prescription (PAP)? A prospective observational analysis of factors that predict increased physical activity
Source: BMC Public Health. 2019 May 2;19:482. doi: 10.1186/s12889-019-6830-1 (PMC6498468; doi:10.1186/s12889-019-6830-1)
Supplement: Supplementary file 5 — Percent of patients with reached PA-level ≥ 5p at 6-month follow-up, analyzed with low vs. high PA at baseline and 1–3 baseline predictive correlates. (PDF 261 kb) [file 12889_2019_6830_MOESM5_ESM.pdf]

Additional file 5 Percent of patients with reached PA-level ≥ 5p at 6-month follow-up, analysed with low vs. high PA at baseline and 1-3 baseline predictive correlates

**Correlate of PA (n)**	**Reached PA-level (≥ 5p)**			
	**% of patients**		***p* value[a]**	**phi coefficient**
	**Low/Positive values**	**High/Negative values**		
PABL/SEE (36/34)	43.9	46.6	0.739	0.02
PABL/PREP (39/33)	42.9	41.8	0.886	0.01
PABL/CONF (38/32)	43.7	40.5	0.679	0.03
PABL/PCS (36/33)	39.1	40.2	0.881	0.01
PABL/BMI (28/48)	43.1	42.1	0.899	0.01
PABL/CONF/PREP (29/19)	44.6	38.0	0.476	0.07
PABL/CONF/PREP/SEE (21/13)	50.0	44.8	0.668	0.05
	**Low/Negative values**	**High/Positive values**		
PABL/SEE (26/39)	31.7	44.3	0.091	0.13
PABL/PREP (28/47)	32.9	51.1	**0.015**	0.18
PABL/CONF (28/48)	31.8	52.2	**0.006**	0.21
PABL/PCS (28/47)	35.0	52.8	**0.020**	0.18
PABL/BMI (41/37)	36.6	56.9	**0.009**	0.20
PABL/CONF/PREP (18/34)	29.0	54.0	**0.005**	0.25
PABL/CONF/PREP/SEE (10/20)	25.6	50.0	**0.026**	0.25

PA-level ≥ 5p physical activity level according to ACSM/AHA questionnaire and public health recommendation, *PABL* physical activity at baseline, *SEE* self-efficacy expectations, *PREP* readiness to change – prepared, *CONF* readiness to change – confident, *PCS* physical component summary - SF-36, *BMI* body mass index
[a]*P* values were determined by Chi-square test for independence
Statistical significance was set at $p \leq 0.05$
